# Common diseases of cattle in Jordan: A retrospective study (2015–2021)

**DOI:** 10.14202/vetworld.2022.2910-2916

**Published:** 2022-12-23

**Authors:** Myassar Alekish, Zuhair Bani Ismail

**Affiliations:** Department of Veterinary Clinical Sciences, Jordan University of Science and Technology, Irbid, Jordan

**Keywords:** animal welfare, cattle diseases, Holstein-Friesian dairy cows, socioeconomic impact of animal disease

## Abstract

**Background and Aim:**

In many developing countries, infectious and non-infectious diseases remain a major hurdle in achieving satisfactory status related to animal health, productivity, and welfare. In Jordan, there are no comprehensive reports describing the most common diseases involving different body systems in different age groups of cattle. Therefore, this retrospective study was designed to report the frequencies of various infectious and non-infectious diseases and their distribution according to sex, age, and body system in cattle in Jordan.

**Materials and Methods:**

Case medical records of cattle presented for clinical evaluation to the Veterinary Health Center of the Faculty of Veterinary Medicine at Jordan University of Science and Technology between January 2015 and December 2021 were used in this study. The data were categorized based on sex (female vs. male), body system involved in the disease process, nature of the disease process (infectious vs. non-infectious), and age (pre-weaning [<2 months of age], 2 months–2 years of age, and older than 2 years of age). Descriptive analysis was performed to report the frequencies, averages, and range values using Excel spreadsheets.

**Results:**

Medical records of 513 cattle cases were used in the study. All cattle belonged to the Holstein-Friesian dairy breed. The majority of cases were female (91%). The age of animals ranged between 1 day and 8 years. According to age groups, there were 52%, 27%, and 16% of cases older than 2 years, 2 months–2 years, and pre-weaning (<2 months), respectively. Among males and females, the majority of cases were diagnosed with gastrointestinal diseases (30%), followed by udder/teat diseases (18%), reproductive and obstetrical diseases (16%), and respiratory diseases (11%). Other body systems involved in disease processes were metabolic (7%), musculoskeletal (6%), cardiovascular/circulatory (4%), multiple systems (3%), nervous (2%), ear/eye (2%), and skin (1%).

**Conclusion:**

Results of this study provide valuable information on the most likely diagnostic list of diseases involving various body systems of different age groups in cattle in Jordan. This information could serve as a clinical guideline for field diagnosis of cattle diseases and provide an accurate estimate of the current status of cattle welfare, health, and husbandry practices in Jordan.

## Introduction

The total cattle population in Jordan is approximately 78,000 head, mostly of dairy breeds [1]. The dairy cattle industry contributes about 14% to the national agriculture product with an approximate total investment of 400–600 million Jordan dinar (JD) [1]. At present, local fresh milk production is estimated to reach 70%–80% of the yearly expected needs of the nation, while cattle contributions to red meat production are shy of 30% [1]. These facts have raised the alarm of the need to improve productivity of livestock in Jordan to reach more stable and sustainable milk and meat supplies to cover expected national needs in the near future.

The status of animal health reflects not only the level of economic and social development but also the level of animal welfare and standards of animal husbandry [2]. In the developed world, major strides have been achieved in veterinary care, animal genetics, animal nutrition, and general farm management that have significantly reduced the impact of animal diseases [3]. In developing countries, however, diseases continue to cause dramatic losses [4].

In Jordan, only scattered scientific research studies could be cited in the literature that highlighted the prevalence of some important infectious animal diseases such as foot and mouth disease [5], brucellosis [6], lumpy skin disease [7], and leptospirosis [8]. Therefore, this retrospective study was conducted to report the frequencies and distribution of infectious and non-infectious diseases in cattle according to age, sex, and affected body system. The results of this study will provide clinical guidelines for field diagnosis of common diseases in cattle and the status of cattle management and husbandry practices in Jordan.

## Materials and Methods

### Ethical approval

Ethical approval was not required in this study as there were no live animals used.

### Study period and location

Case medical records of all cattle presented for clinical evaluation to the Veterinary Health Center (VHC) of the Faculty of Veterinary Medicine at Jordan University of Science and Technology from 2015 to 2021 were used in this study. The VHC is a mixed animal outpatient practice located in North Jordan that provides tertiary veterinary care to various animal species within a 50 km radius. Jordan is located in the center of the Middle East between Lat. 29° 30’ and 32° 31’. The northern regions are mountainous in nature with an average annual rainfall of 400–600 mm [9]. Cattle in this region of Jordan typically belong to smallholder family-owned farms and are raised in traditional farming conditions. Animals are allowed to graze on available grasses and fodder during the spring and totally rely on grain-based concentrate diets the rest of the year [10].

### Case selection and data collection

To be included in the study, the case medical record must have been completed with the basic patient identification data, including animal species, breed, age, and sex, and medical data including the presenting complaint, pertinent history relevant to the farm and diseased individual animal, physical examination findings, laboratory findings, clinical diagnosis, and medical or surgical treatment recommended for the animal. Case medical records with missing data were excluded from the analysis. Systemic viral and septicemic bacterial infections were classified as conditions affecting multiple systems in this study. Animals that were presented with clinical signs suggestive of respiratory infections such as fever, coughing, nasal and mucopurulent nasal discharge, and abnormal pulmonary sounds on auscultation with no other abnormalities involving any other body system were diagnosed with bronchopneumonia regardless of the possible etiologic nature (bacterial, viral, or parasitic). Although bacterial culture of milk from cases of clinical mastitis cases is routinely performed, specific bacterial etiologic diagnosis was not reported in this study and the term “bacterial mastitis” was used for all cases of mastitis reported in this study.

### Statistical analysis

Data were extracted from the case medical records and tabulated using Microsoft Excel spreadsheets (Microsoft Word 10, Microsoft Co., USA). The data were categorized based on sex (female vs. male), body system involved in the disease process, and nature of the disease process (infectious vs. non-infectious), and age (pre-weaning [<2 months of age], 2 months–2 years of age, and older than 2 years of age). Descriptive analysis was performed to report the frequencies, averages, and range values.

## Results

The categorical data of all cases included in the study are presented in Table-1. Case records of 513 animals were used in this retrospective study. All cattle belonged to the Holstein-Friesian dairy breed. The age of animals ranged between 1 day and 8 years. According to age groups, there were 52%, 27%, and 16% of cases older than 2 years, 2 months–2 years of age, and pre-weaning (<2 months of age), respectively.

**Table-1:** Categorical data of cattle cases presented for veterinary medical evaluation in North Jordan from 2015 to 2021 (n = 513).

Category	Number of cases	Percentage
Sex		
Female	467	91
Male	46	9
Age (range 1–8 years)		
Pre-weaning (≤2 months)	80	16
>2 months–≤2 years	137	27
>2 years	269	52

According to sex, the majority of cases were female (91%). The most commonly diagnosed diseases in males involved the gastrointestinal system (41.3%), followed by the respiratory system (32.6%), musculoskeletal system (10.9%), cardiovascular system (6.5%), eye/ear (4.35%), and metabolic diseases and nervous system (2.18% each).

**Figure-1:**
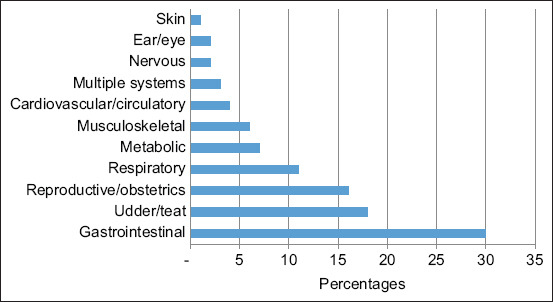
Distribution of diseases (%) involving various body systems in cattle (n = 513).

The distribution of diseases according to age and body system in cattle is presented in Figure-1 and Table-2. About 30% of cattle were diagnosed with gastrointestinal diseases (30%). The majority of pre-weaning age calves were diagnosed with enteritis (49%) and abomasitis (7.5%). In cattle aged 2 months–2 years, the most common gastrointestinal diseases were simple indigestion (11%) and displaced abomasum (6.5%) while in cattle older than 2 years, the most common gastrointestinal diseases were displaced abomasum (7.5%), simple indigestion (6.5%), rumen tympany (4.5%), enteritis (4%), and vagal indigestion (3.5%).

**Table-2:** Distribution of diseases (absolute numbers) involving various body systems categorized according to the age in cattle (n = 513).

System involved (N)	Pre-weaning (<2 months) (n = 80)	≥2 months–≤2 years (n = 137)	>2 years (n = 296)
Gastrointestinal (157)	Enteritis (39)	Simple indigestion (15)	Displaced abomasum (22)
	Abomasitis (6)	Displaced abomasum (9)	Simple indigestion (19)
		Enteritis (3)	Rumen tympany (13)
		Pharyngitis (3)	Enteritis (11)
			Vagal indigestion (10)
			Pharyngitis (5)
			Cecal dilatation (2)
Udder/teat (92)	-	Mastitis (14)	Mastitis (55)
		Udder edema (5)	Udder edema (13) Teat injuries (5)
Reproductive/obstetrics (82)	-	Abortion (13)	Abortion (24)
		Dystocia (5)	Repeat breeder (19)
		Retained placenta (4)	Metritis (11)
		Metritis (3)	Retained placenta (3)
Respiratory (55)	Bronchopneumonia (14)	Bronchopneumonia (19)	Bronchopneumonia (22)
Metabolic (35)	White muscle disease (2)	Ketosis (9)	Ketosis (15)
		Hypocalcemia (2)	Hypocalcemia (6)
Musculoskeletal (30)	Fractures (4) Polyarthritis (7)	Septic arthritis (1)	Foot rot (15) Septic arthritis (3)
Multiple systems (15)	Septicemic salmonellosis (2)	Bovine viral diarrhea virus (5) Enterotoxemia (6)	Bovine viral diarrhea virus (2)
Nervous (12)	Bacterial meningitis (6)	Parturient paresis (3)	Nervous ketosis (3)
Cardiovascular (21)	-	Traumatic pericarditis (9)	Traumatic pericarditis (12)
Ear/eye (10)	-	Keratoconjunctivitis (5) Otitis (2)	Squamous cell carcinoma (3)
Skin (5)	-	Wound (2)	Mange (2) Wound (1)

Diseases involving the udder and teats were the second most commonly diagnosed conditions in cattle (18%). Acute clinical mastitis was diagnosed in 19% and 10% of cattle aged older than 2 years and cattle aged 2 months–2 years, respectively. Udder edema was diagnosed in 4.4% and 3.6% of cattle aged older than 2 years and cattle aged 2 months–2 years, respectively. Teat lacerations were diagnosed in 1.7% of cattle aged older than 2 years.

Diseases involving the reproductive system were the third most commonly diagnosed conditions in cattle (16%). Abortion (8%), repeat breeders (6.5%), metritis (3.7%), and retained placenta (1%) were the most common diseases diagnosed in cattle aged older than 2 years, while in heifers up to 2 years of age, abortion (9.5%), dystocia (3.6%), retained placenta (3%), and metritis (2.2%) were the most commonly diagnosed reproductive conditions.

About 11% of cattle were diagnosed with respiratory diseases. A diagnosis of bronchopneumonia caused by unidentified (bacterial, viral, or parasitic etiology) was made in 17.5%, 13.8%, and 7.4% of cases in pre-weaning age, cattle aged 2 months–2 years, and cattle aged older than 2 years, respectively.

Metabolic diseases were diagnosed in 7% of cattle in this study. The highest number of metabolic diseases was diagnosed in cattle aged 2 months–2 years and those aged older than 2 years. Ketosis was the most commonly diagnosed condition in cattle aged 2 months–2 years and those aged older than 2 years (6.5% and 5%, respectively) while in pre-weaning age calves, white muscle disease was diagnosed in 2.5% of the cases. Hypocalcemia was diagnosed in 2% and 1.5% of cattle older than 2 years and those 2 months–2 years, respectively.

Diseases involving the musculoskeletal system were diagnosed in 6% of cattle in the study. Foot rot (5%) and polyarthritis (8.8%) were the most commonly diagnosed musculoskeletal conditions in cattle aged older than 2 years and pre-weaning calves, respectively. Fractures were diagnosed in 5% of pre-weaning calves. Septic arthritis was diagnosed in 1% and 0.7% of cattle aged older than 2 years and those aged 2 months–2 years, respectively.

Diseases involving the cardiovascular/circulatory systems were diagnosed in 4% of cattle. Traumatic pericarditis was diagnosed in cattle aged 2 months–2 years (6.6%) and those aged older than 2 years (4%).

Diseases characterized by multiple systems manifestations were diagnosed in 3% of cattle. The most common diseases in this category were viral infection with bovine viral diarrhea virus, enterotoxemia caused by *Clostridium* spp., and septicemic salmonellosis.

Diseases involving the nervous system were diagnosed in 2% of the cases. Meningitis (7.5%) in pre-weaning calves, parturient paresis (2.2%) in cattle aged 2 months–2 years, and nervous ketosis (1%) in cattle aged older than 2 years were the most commonly diagnosed nervous diseases in this study.

Diseases of the eyes and ears were diagnosed in 2% of the cases. Infectious keratoconjunctivitis (3.6%) and otitis (1.5%) were the most commonly diagnosed diseases in cattle aged 2 months–2 years while squamous cell carcinoma (1%) was diagnosed in cattle aged older than 2 years.

Skin diseases were diagnosed in 1% of the cases. Traumatic skin wounds and mange were the most commonly diagnosed conditions of the skin in cattle.

The distribution of infectious diseases involving various body systems in cattle is presented in Table-3. In the gastrointestinal system, the most commonly diagnosed infectious causative agents were coronavirus, *Mycobacterium paratuberculosis*, *Salmonella* spp., *Eimeria* spp., *Cryptosporidium parvum*, and various species of intestinal parasites (Nematode spp.). In the reproductive system, infectious agents causing abortion were very common, including *Brucella* spp., *Toxoplasma gondii*, and *Neospora caninum*. Infectious agents with multiple systems manifestations were bovine viral diarrhea virus, *Clostridium* spp., and *Salmonella* spp. Moraxella bovis was the causative agent of infectious keratoconjunctivitis and *Mycoplasma* spp. was the cause of otitis media/interna in cattle in this study.

**Table-3:** The most common infectious diseases categorized according to the body system involved in cattle.

Systems involved	Diagnosis	Infectious agent
Gastrointestinal	Enteritis	Corona virus *Cryptosporidium parvum* *Mycobacterium paratuberculosis* *Eimeria* spp. Intestinal parasites: Nematodes
Reproductive	Abortion	*Brucella* spp. *Toxoplasma gondii* *Neospora caninum*
Multiple systems	Bovine viral diarrhea	Pestivirus
	Enterotoxemia	*Clostridium* spp.
	Septicemia	*Salmonella* spp.
Eye	Keratoconjunctivitis	*Moraxella bovis*

## Discussion

In this study, the frequencies of disease conditions affecting cattle of various sex and ages in North Jordan were determined. Although the study population is confirmed of cases presented to one VHC located in the northern region of Jordan, it fairly represents the cattle population in the region. Cattle in Jordan are mostly of the dairy breed (Holstein-Friesians). Female calves are typically selected for replacement as early as 2 months of age while males are destined for dairy-beef production as early as 1 week of age. This was accurately depicted in the study population as 91% of the cases were female and only 9% were male.

The top five systems involved in disease processes in cattle in this study were gastrointestinal, udder/teats, reproductive/obstetrics, respiratory, and metabolic systems. Worldwide, these diseases are considered the cause of major economic losses in dairy farms [11]. In pre-weaning calves, infectious enteritis caused by viral agents (coronavirus), protozoan agents (*C. parvum*), and *Eimeria* spp. was the most frequently diagnosed disease. Enteritis remains the leading cause of mortality in neonatal calves [12]. Enteritis is characterized by diarrhea, dehydration, metabolic acidosis, and toxemia, leading to rapid deterioration and death in neonatal calves [13]. Significant risk factors for neonatal diarrhea are maternal nutrition, calf hood vaccination, maternity pen hygiene and calving practices, and colostrum management [14, 15].

The second most common disease diagnosis in pre-weaning calves in this study was bronchopneumonia. Bronchopneumonia is a complex disease with significant lifelong health effects in calves [16, 17]. Etiologically, pneumonia is typically initiated by poor housing and environmental conditions, leading to damaged mucous membranes of the respiratory system [16, 17]. Several viruses such as bovine herpesvirus 1, bovine respiratory syncytial virus, and parainfluenza 3 virus and many strains of bacteria such as *Mycoplasma* spp., *Pasteurella multocida*, *Mannheimia haemolytica*, and *Histophilus somni* are responsible for severe manifestations of bronchopneumonia [16, 17]. In this study, an etiological causative agent could not be reported because such an etiological diagnosis of pneumonia is often not sought unless it was a part of a herd outbreak investigation.

In heifers aged between 2 months and 2 years and cows older than 2 years, the most commonly diagnosed diseases involved the gastrointestinal system, followed by the udder and teats, reproductive and respiratory systems. In the gastrointestinal system, the majority of cases were diagnosed with simple indigestion, displaced abomasum, and rumen tympany. These diseases are considered common in dairy cows in early lactation due to poor nutritional management [18]. Lack of adequate quality forages and poor transitional management are the culprits for these important diseases in dairy cattle [18, 19]. Vagal indigestion is a neurofunctional disorder of the upper gastrointestinal tract of cattle resulting in progressive gastric dilation and eventually death [20]. This disease is often end-stage and most frequently associated with chronic inflammatory conditions such as peritonitis, peritoneal and liver abscesses, and tumors involving the gastrointestinal tract such as lymphosarcoma and fibropapilloma [21].

Clinical mastitis was the second most common disease diagnosis in cattle aged 2 months–2 years and those older than 2 years. Common mastitis pathogens in Jordan’s dairy cows have been reported previously, and therefore, the etiological agents of mastitis are not reported in this study [22]. Mastitis is considered one of the most economically important diseases in dairy cattle industries worldwide [23]. In dairy farms, increased incidence of clinical mastitis is a direct measure of the cow’s health, stall hygiene, and comfort and parlor operation [15, 24]. Mastitis rates could be reduced by applying strict control measures, including segregation and treatment of clinical cases, adaptation of proper milking procedures, teat disinfection, dry cow therapy, vaccination, and improved environmental hygiene [25].

Udder edema was the second most common condition diagnosed in the udder in cows in this study. The condition has been gaining more recognition in recent years due to a steady rise in its prevalence in dairy farms and its impact on animal productivity and welfare [26, 27]. Udder edema is caused by physiological udder swelling causing pain, reduced milk production, production of unsalable milk due to the presence of blood cells, and increased susceptibility to mastitis. Recent study has suggested a potential causative relationship between udder edema and liver dysfunction associated with a dietary deficiency in dry matter and excessive intake of sodium and potassium in the pre-partum diet [28].

Diseases involving the reproductive system and obstetrical conditions were the third most common diseases reported in this study. Abortion accounted for 9.4% and 8% of cases in heifers and in cows older than 2 years, respectively. Abortion is considered one of the most economically important conditions in cattle practice [29]. Diagnosis of abortion still presents a major challenge in the field as well as in the diagnostic laboratory [29]. In this study, although some infectious causative etiologies could have been identified, in the majority of abortion cases, an etiological agent could not. These results are similar to previously published data which indicated that <50% of causative etiologies of cattle abortions could be identified [30]. In this study, the most common infectious causes of abortion were *Brucella* spp., *T. gondii*, and *N. caninum*. Similarly, recent studies have suggested that protozoal infections, namely, neosporosis, were the most commonly diagnosed cause of abortion in cattle.

In heifers, dystocia was the second most frequently diagnosed condition involving the reproductive system while in older cows, repeat breeder followed by metritis was the second and third most common conditions, respectively. Dystocia is a serious condition that could result with the death of the cow or the calf or both [31]. In addition, calves and cows require special care following dystocia, adding to the cost and production losses associated with dystocia. Dystocia is more frequently observed in dairy heifers due to fetal-pelvic disproportion while in older cows, dystocia is likely caused by fetal malposition [31]. Dealing with dystocia successfully requires adequate training and proper and early intervention to ensure favorable outcomes [31]. However, prevention requires increased efforts to genetically select cows for calving ease, improving heifer nutrition and rearing conditions to ensure adequate growth [31].

Repeat breeding is another important and common cause of reproductive failure and economic losses in dairy farms. Cows affected with repeat breeding fail to conceive after three insemination attempts without obvious anatomical, inflammatory, or infectious abnormalities [32]. The condition is multifactorial, but recent studies have suggested environmental stress, hormonal disturbances, and poor oocyte quality as likely etiological factors [32]. Other related risk factors that may play a role in the occurrence of repeat breeders are age of the cow, nutritional management of the herd, body condition score of the cow, and milk yield [33].

Metritis is a common infectious disease of the uterus in the postpartum period in dairy cows. Metritis may result in significant economic losses due to treatment and veterinary costs, production losses, premature culling, or death of the affected cow [34]. The condition is associated with calving difficulties, retained fetal membranes, and poor nutritional management in the transitional period [34].

In the metabolic system, ketosis and hypocalcemia were the most commonly diagnosed diseases in heifers and older cows. Ketosis is caused by a lack of adequate energy supply in early lactation to support large milk production resulting in gradual loss of body weight, reduced milk production, reduced reproductive functions, and increased risk of other illnesses, such as fatty liver, displaced abomasum, and metritis [35]. Poor nutritional management in the transitional period and postpartum diseases such as mastitis or metritis are common predisposing causes of ketosis in dairy cows [35].

Hypocalcemia is another metabolic and nutritional problem, commonly recognized in older cows. Clinical hypocalcemia is considered a major health and welfare issue in dairy farms [36]. It is characterized by failure of calcium hemostasis and severe hypocalcemia at calving, leading to a significant increase in the incidence of health problems such as gastrointestinal atony, reduced milk production, poor reproductive performance, and, in severe cases, death of the cow [36]. The condition can be prevented by acidification of the pre-partum diet [36].

Other less frequently encountered disease conditions in cattle in this study involved the musculoskeletal (6%), cardiovascular/circulatory (4%), nervous systems (2%), ear/eye (2%), and skin (1%). These diseases could significantly impact the health, productivity, and welfare of individual animals, and in certain situations, it may result in significant economic losses in some farms. However, in this study, most of these diseases are dealt within farms by local veterinarians without the need to seek advanced veterinary consultation.

## Conclusion

This is the first study to provide a comprehensive view of the most commonly diagnosed diseases and their distribution according to etiology (infectious vs. non-infectious, age, and body system) in cattle in Jordan. The top body systems involved in diseases process in cattle were the gastrointestinal, udder/teats, reproductive/obstetrics, respiratory, metabolic, and musculoskeletal systems and those with multiple system manifestations. These results indicate that despite major national efforts in preventative animal health, nutrition, and animal management measures, still disease – both infectious and non-infectious – presents a major hurdle in the path to achieve satisfactory status related to animal health, productivity, and welfare in cattle in Jordan. Specific measures are in demand to improve nutritional management, husbandry practices including biosecurity implementation in cattle farms, revisiting routine farm vaccination, and deworming programs.

## Authors’ Contributions

MA: Conceived and designed the study, collected the data from the case medical records, and reviewed the manuscript. ZBI: Performed data analysis, interpreted the data, and wrote the manuscript. Both authors have read and approved the final manuscript.
